# Supplementary material for: Whole Genome Analysis of 132 Clinical Saccharomyces cerevisiae Strains Reveals Extensive Ploidy Variation
Source: G3 (Bethesda). 2016 Jun 13;6(8):2421–34. doi: 10.1534/g3.116.029397 (PMC4978896; doi:10.1534/g3.116.029397)
Supplement: Supplemental Material [file supp_6_8_2421__index.html]

Whole Genome Analysis of 132 Clinical Saccharomyces cerevisiae Strains Reveals Extensive Ploidy Variation — Supplemental Material 

# Whole Genome Analysis of 132 Clinical *Saccharomyces cerevisiae* Strains Reveals Extensive Ploidy Variation

## Supplemental Material for Zhu, Sherlock, and Petrov, 2016

**Files in this Data Supplement:**

- Figure S1 - Correlation between allele frequencies in non-clinical strains (X-axis) to allele frequencies in clinical strains (Y-axis). (.pdf, 197 KB)
- Figure S2 - Fst values (Y-axis) for alleles across the genome (X-axis). (.pdf, 192 KB)
- Figure S3 - Coverage plots for YJM264 across *S. cerevisiae* and *S. kudriavzevii* genomes. (.pdf, 192 KB)
- Figure S4 - Coverage plots for CBS2909 (a) and CBS2910 (b) across *S. cerevisiae* and *S. paradoxus* genomes. (.pdf, 195 KB)
- Table S1 - List of all strains included in this study. (.pdf, 180 KB)
- Table S2 - List of all fragments thought to be introgressed in strains CBS2909 and CBS2910, and genes that fall within these regions. (.pdf, 220 KB)
- Table S4 - Lists of all genes that showed complete deletion (.pdf, 29 KB)
- Table S5 - List of all genes that showed copy number gain. (.pdf, 57 KB)
- Table S6 - List of all genes that showed copy number loss. (.pdf, 61 KB)
- Table S3 - Tab-delimited file of % reads mapping to each chromosome across 5 reference panel species. (.txt, 248 KB)
