# Supplementary material for: Whole Genome Analysis of 132 Clinical Saccharomyces cerevisiae Strains Reveals Extensive Ploidy Variation
Source: G3 (Bethesda). 2016 Jun 13;6(8):2421–34. doi: 10.1534/g3.116.029397 (PMC4978896; doi:10.1534/g3.116.029397)
Supplement: Supplemental Material [file supp_g3.116.029397_FigureS1.pdf]

Figure S1

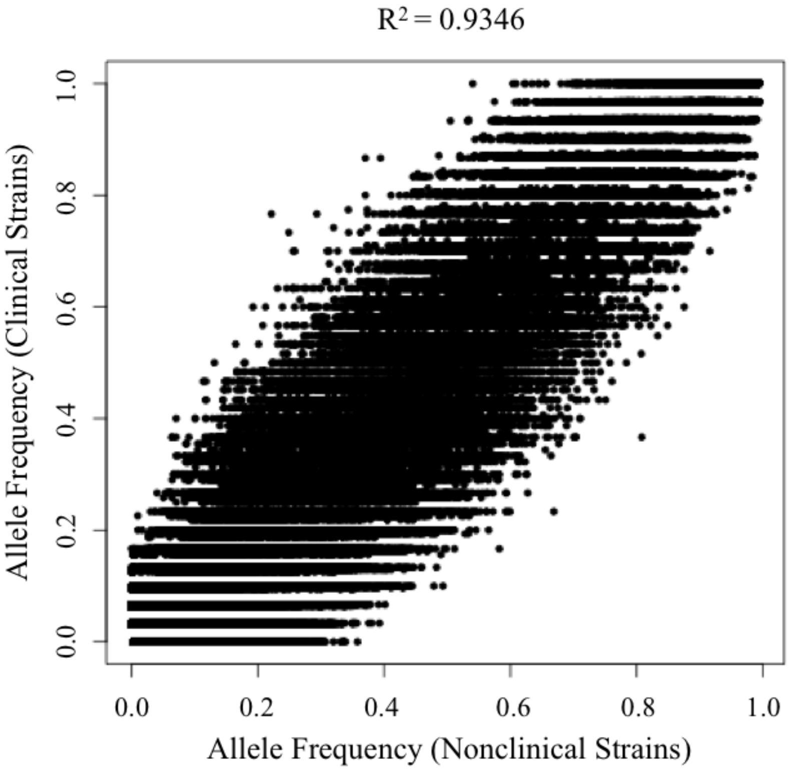

Figure S1. Correlation between allele frequencies in non-clinical strains (X-axis) to allele frequencies in clinical strains (Y-axis).
