# Supplementary figures and images for: Whole Genome Analysis of 132 Clinical Saccharomyces cerevisiae Strains Reveals Extensive Ploidy Variation
Source: G3 (Bethesda). 2016 Jun 13;6(8):2421–34. doi: 10.1534/g3.116.029397 (PMC4978896; doi:10.1534/g3.116.029397)

Figure S2

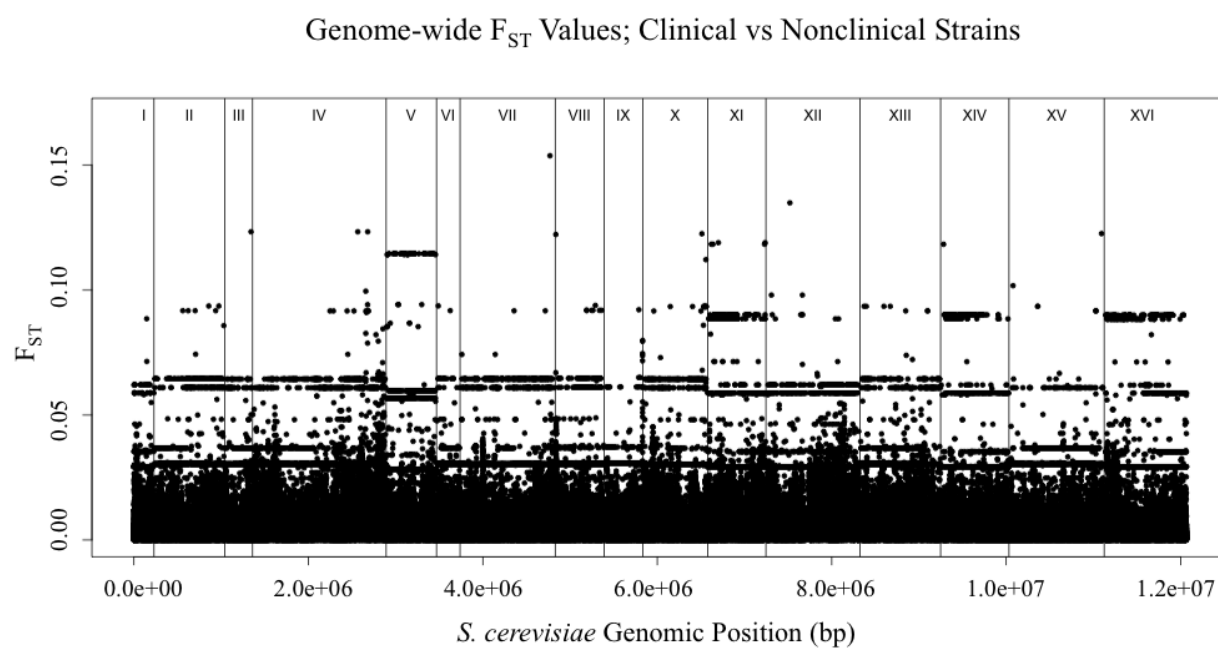

Figure S2.  $F_{ST}$  values (Y-axis) for alleles across the genome (X-axis).

Supplement: Supplemental Material [file supp_g3.116.029397_FigureS2.pdf]
