# Supplementary material for: Whole Genome Analysis of 132 Clinical Saccharomyces cerevisiae Strains Reveals Extensive Ploidy Variation
Source: G3 (Bethesda). 2016 Jun 13;6(8):2421–34. doi: 10.1534/g3.116.029397 (PMC4978896; doi:10.1534/g3.116.029397)
Supplement: Supplemental Material [file supp_g3.116.029397_FigureS3.pdf]

Figure S3

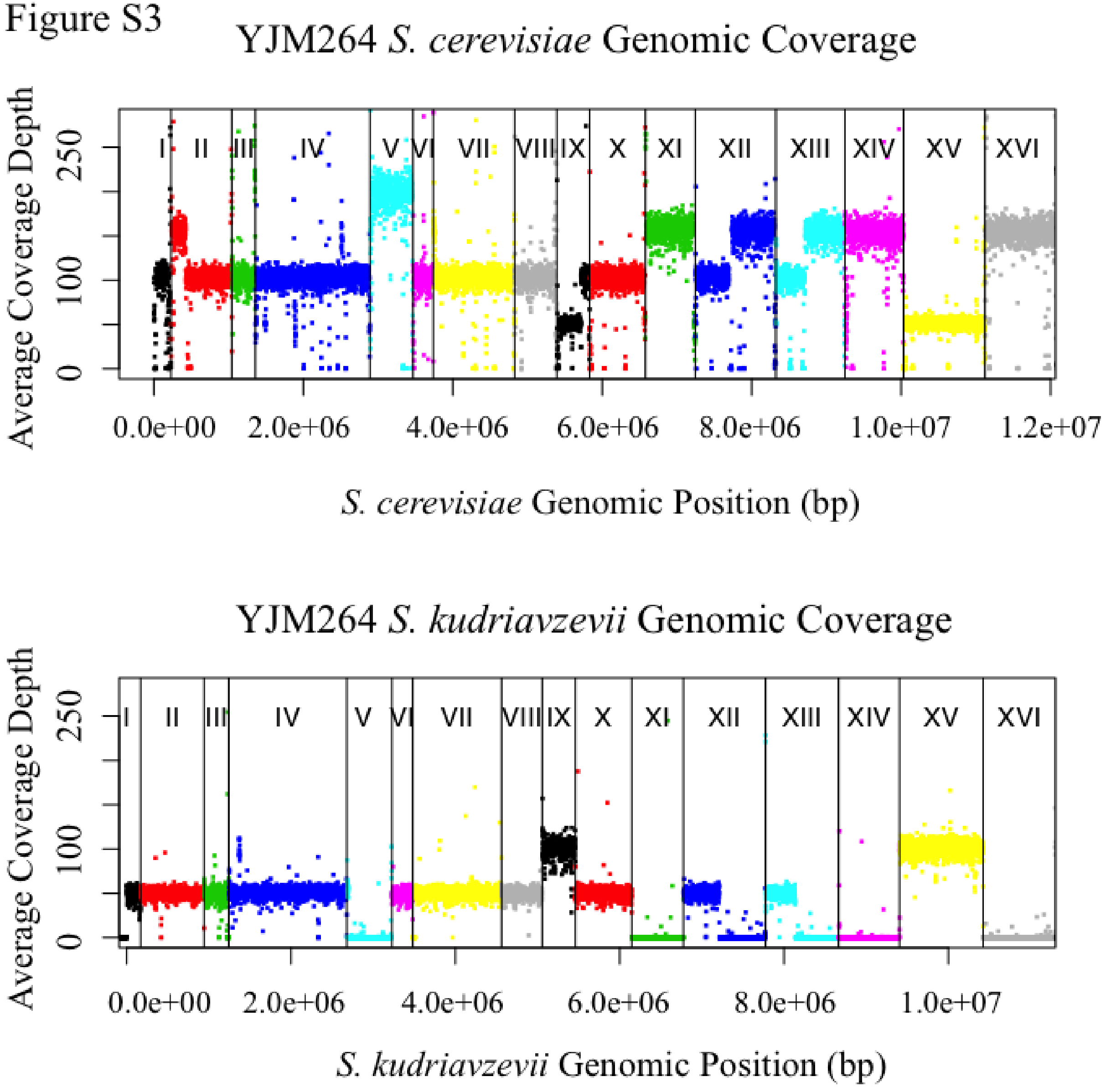

Figure S3. Coverage plots for YJM264 across *S. cerevisiae* and *S. kudriavzevii* genomes. Chromosomes are lined in increasing order (X-axis), with total coverage in 1kb windows plotted (Y-axis).
