# Supplementary material for: Whole Genome Analysis of 132 Clinical Saccharomyces cerevisiae Strains Reveals Extensive Ploidy Variation
Source: G3 (Bethesda). 2016 Jun 13;6(8):2421–34. doi: 10.1534/g3.116.029397 (PMC4978896; doi:10.1534/g3.116.029397)
Supplement: Supplemental Material [file supp_g3.116.029397_FigureS4.pdf]

Figure S4a

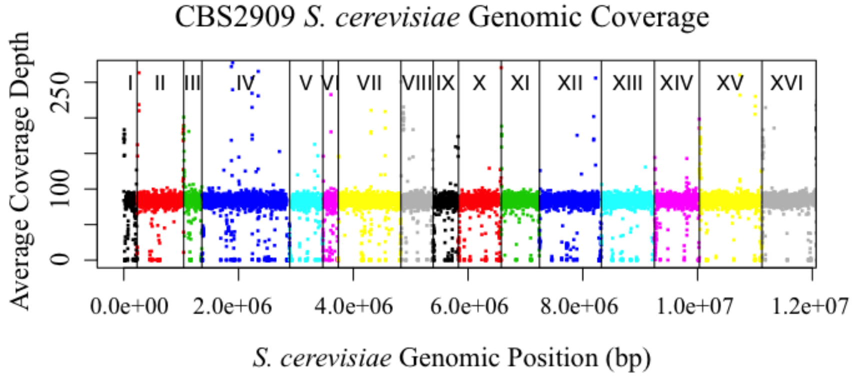
CBS2909 *S. cerevisiae* Genomic Coverage
Average Coverage Depth
0
100
250
I
II
III
IV
V
VI
VII
VIII
IX
X
XI
XII
XIII
XIV
XV
XVI
0.0e+00
2.0e+06
4.0e+06
6.0e+06
8.0e+06
1.0e+07
1.2e+07
S. cerevisiae Genomic Position (bp)

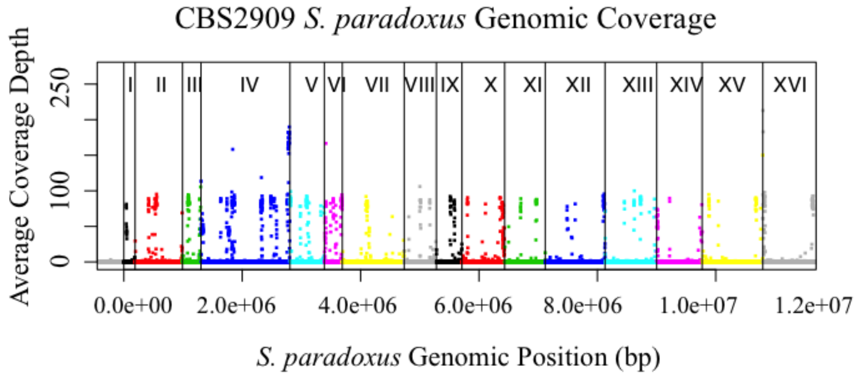
CBS2909 *S. paradoxus* Genomic Coverage
Average Coverage Depth
0
100
250
I
II
III
IV
V
VI
VII
VIII
IX
X
XI
XII
XIII
XIV
XV
XVI
0.0e+00
2.0e+06
4.0e+06
6.0e+06
8.0e+06
1.0e+07
1.2e+07
S. paradoxus Genomic Position (bp)

Figure S4b

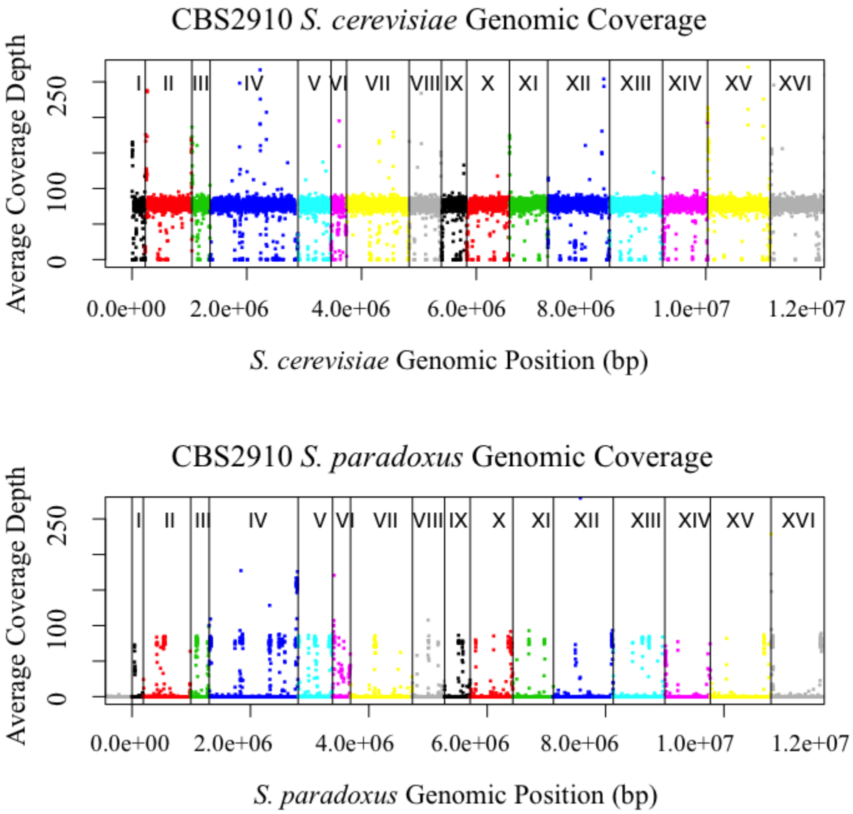

Figure S4. Coverage plots for CBS2909 (a) and CBS2910 (b) across *S. cerevisiae* and *S. paradoxus* genomes. Chromosomes are lined in increasing order (X-axis), with total coverage in 1kb windows plotted (Y-axis).
