# Supplementary material for: Whole Genome Analysis of 132 Clinical Saccharomyces cerevisiae Strains Reveals Extensive Ploidy Variation
Source: G3 (Bethesda). 2016 Jun 13;6(8):2421–34. doi: 10.1534/g3.116.029397 (PMC4978896; doi:10.1534/g3.116.029397)
Supplement: Supplemental Material [file supp_g3.116.029397_TableS1.pdf]

Table S1 - List of all strains included in this study.

Tissue Name / Source	Individual Name	MapID	Strain	Reference		
Clinical	08-117	2-018	YJM223	Clemons 94		de llanos 06
Fleischmann’s baking yeast	08-118	2-019	YJM263	Clemons 94		
Red Star baking yeast	08-119	2-020	YJM264	Clemons 94	MullerMcC 09	
Clinical	08-120	2-021	YJM432			
Clinical	08-121	2-022	B68019c		MullerMcC 09	
Clinical	08-122	2-023	YJM435			
Clinical	08-123	2-024	YJM436	Clemons 94	MullerMcC 09	
Clinical	08-124	2-025	B68549		MullerMcC 09	
Clinical	08-125	2-026	B66044		MullerMcC 09	
Clinical	08-126	2-027	YJM439			
Clinical	08-127	2-028	92-123	Clemons 94	MullerMcC 09	
Clinical	08-128	2-029	YJM455	Clemons 94	MullerMcC 09	
Clinical	08-129	2-030	YJM464			
Clinical	08-131	2-032	YJM466			
Clinical	08-132	2-033	YJM467			
Clinical	08-133	2-034	YJM521			
Clinical	08-134	2-035	YJM525		MullerMcC 09	
Clinical	08-135	2-036	YJM560			
Clinical	08-136	2-037	YJM561			
Clinical	08-137	2-038	YJM634			
Clinical	08-138	2-039	YJM669			
Clinical	08-139	2-040	YJM670			
Clinical	08-140	2-041	YJM671			
Clinical	08-141	2-042	YJM672			
Clinical	08-142	2-043	YJM674			
Clinical	08-143	2-044	YJM676			

Clinical	08-144	2-045	YJM677	MullerMcC 09
Clinical	08-145	2-046	YJM678	
Clinical	08-146	2-047	YJM810	
Clinical	08-147	2-048	YJM811	
Clinical	08-148	2-049	YJM813	
Clinical	08-149	2-050	YJM815	
Clinical	08-150	2-051	YJM816	
Clinical	08-151	2-052	YJM936	
Clinical	08-152	2-053	YJM945	MullerMcC 09
Clinical	08-153	2-054	YJM946	MullerMcC 09
Clinical	08-154	**2-055**	YJM947	MullerMcC 09
Clinical	08-155	**2-056**	YJM948	MullerMcC 09
Clinical	08-156	2-057	YJM949	MullerMcC 09
Clinical	08-157	2-058	YJM950	MullerMcC 09
Clinical	08-158	2-059	YJM951	MullerMcC 09
Clinical	08-159	2-060	YJM952	MullerMcC 09
Clinical	08-160	2-061	YJM953	MullerMcC 09
Clinical	08-161	**2-062**	YJM954	MullerMcC 09
Clinical	08-162	**2-063**	YJM955	MullerMcC 09
Clinical	08-163	**2-064**	YJM956	MullerMcC 09
Clinical	08-164	**2-065**	YJM957	MullerMcC 09
Commercial	08-165	2-066	YJM958	MullerMcC 09
Commercial	08-166	2-067	YJM959	MullerMcC 09
Commercial	08-167	2-068	YJM960	MullerMcC 09
Commercial	08-168	2-069	YJM961	MullerMcC 09
Clinical	08-169	2-070	YJM962	MullerMcC 09
Clinical	08-170	**2-071**	YJM963	MullerMcC 09
Clinical	08-171	**2-072**	YJM964	MullerMcC 09

Clinical	08-172	**2-073**	YJM965	MullerMcC 09
Clinical	08-173	2-074	YJM966	MullerMcC 09
Clinical	08-174	2-075	YJM967	MullerMcC 09
YJM #1004	08-175	2-076	S288C	MullerMcC 09
YJM #1005	08-176	2-077	YJM1005	MullerMcC 09
YJM #1006	08-177	2-078	YJM1006	MullerMcC 09
clinical	08-178	2-079	YJM1094	MullerMcC 09
clinical	08-179	2-080	YJM1095	MullerMcC 09
clinical	08-180	2-081	YJM1096	MullerMcC 09
clinical	08-181	2-082	YJM1097	MullerMcC 09
clinical	08-182	2-083	YJM1098	MullerMcC 09
clinical	08-183	2-084	YJM1099	MullerMcC 09
clinical	08-184	2-085	YJM1100	MullerMcC 09
clinical	08-185	2-086	YJM1101	MullerMcC 09
clinical	08-186	2-087	YJM1102	MullerMcC 09
clinical	08-187	2-088	YJM1103	MullerMcC 09
clinical	08-188	2-089	YJM1104	MullerMcC 09
clinical	08-189	2-090	YJM1105	MullerMcC 09
clinical	08-190	2-091	YJM1106	MullerMcC 09
clinical	08-191	2-092	YJM1107	MullerMcC 09
clinical	08-192	2-093	YJM1108	MullerMcC 09
clinical	08-193	2-094	YJM1109	MullerMcC 09
clinical	08-194	2-095	YJM1110	MullerMcC 09
clinical	08-195	2-096	YJM1111	MullerMcC 09
clinical	08-196	2-097	YJM1112	MullerMcC 09
clinical	08-197	2-098	YJM1113	MullerMcC 09
clinical	08-198	2-099	YJM1114	MullerMcC 09
clinical	08-199	2-100	YJM1115	MullerMcC 09

clinical	08-200	2-101	YJM1116		MullerMcC 09	
clinical	08-201	2-102	YJM1117		MullerMcC 09	
clinical	08-202	2-103	YJM1118		MullerMcC 09	
clinical	08-203	2-104	YJM1119		MullerMcC 09	
clinical	08-204	2-105	YJM1120		MullerMcC 09	
clinical	08-205	2-106	YJM1121		MullerMcC 09	
clinical	08-206	2-107	YJM1122		MullerMcC 09	
clinical	08-207	2-108	YJM1135		MullerMcC 09	
Unknown	08-213	2-114	YJM1292			
Segregant of clinical isolate YJM128	09-109	2-115	YJM145	Clemons 94		de llanos 06
Segregant of non-clinical isolate CBS 2808	09-110	2-116	YJM269			
Segregant of non-clinical isolate CBS 2807	09-111	2-117	YJM270			
Segregant of clinical isolate	09-112	2-118	YJM280			
Clinical isolate	09-113	2-119	YJM308	Clemons 94		
Segregant of clinical isolate	09-114	2-120	YJM320			Muller 11
Segregant of clinical isolate	09-115	2-121	YJM326			Muller 11
Non-clinical, original name UCD91-1, obtained from L. Bisson	09-116	2-122	YJM332	Clemons 94	MullerMcC 09	de llanos 06
Segregant of clinical isolate	09-117	2-123	YJM339			Muller 11
Segregant of clinical isolate	09-118	2-124	YJM421			
Clinical isolate, original name B68019c; obtained from K. Clemons/D. Stevens who obtained it from F. Odds	09-119	2-125	YJM434		MullerMcC 09	
Clinical isolate, original name B70302(b); obtained from K. Clemons/D. Stevens who obtained it from F. Odds	09-120	2-126	B70302b	Clemons 94	MullerMcC 09	
Clinical isolate, original name 92-123, obtained from K. Clemons/D. Stevens	09-121	2-127	YJM440	Clemons 94	MullerMcC 09	
Clinical isolate, original name 89-156, obtained from K. Clemons/D. Stevens	09-122	2-128	YJM454	Clemons 94	MullerMcC 09	
Nonclinical; isogenic with/segregant of Y55	09-123	2-129	YJM627			Muller 11

Haploid derivative of/isogenic to YJM145	09-124	2-130	YJM789	Clemons 94		de llanos 06
Clinical isolate, original name MMRL124; obtained from W. Schell/T. Mitchell	09-125	2-131	YJM1124		MullerMcC 09	
Clinical isolate, original name MMRL125; obtained from W. Schell/T. Mitchell	09-126	2-132	YJM1125		MullerMcC 09	
Non-clinical, segregant of NRRL Y-53 (=NRRL Y-567)	09-127	2-133	YJM1129			Muller 11
Clinical isolate, original name MMRL 2138; obtained from W. Schell/T. Mitchell	09-128	2-134	YJM1138		MullerMcC 09	
Clinical isolate, original name MMRL 2195; obtained from W. Schell/T. Mitchell	09-129	2-135	YJM1139		MullerMcC 09	
Clinical isolate, original name MMRL 2416; obtained from W. Schell/T. Mitchell	09-130	2-136	YJM1140		MullerMcC 09	
Clinical isolate, original name MMRL 2297; obtained from W. Schell/T. Mitchell	09-131	2-137	YJM1141		MullerMcC 09	
Clinical isolate, original name MMRL 2218; obtained from W. Schell/T. Mitchell	09-132	2-138	YJM1142		MullerMcC 09	
Clinical isolate, original name MMRL 2236; obtained from W. Schell/T. Mitchell	09-133	2-139	YJM1143		MullerMcC 09	
Clinical isolate, original name MMRL 2237; obtained from W. Schell/T. Mitchell	09-134	2-140	YJM1144		MullerMcC 09	
Clinical isolate, original name MMRL 2244; obtained from W. Schell/T. Mitchell	09-135	2-141	YJM1145		MullerMcC 09	
Clinical isolate, original name MMRL 2248; obtained from W. Schell/T. Mitchell	09-136	2-142	YJM1146		MullerMcC 09	
Clinical isolate, original name MMRL 2497; obtained from W. Schell/T. Mitchell	09-137	2-143	YJM1178		MullerMcC 09	
Clinical isolate	09-138	2-144	YJM1259		MullerMcC 09	
Clinical isolate; obtained from J. Bakken	09-139	2-145	YJM1289			
infected nail, of 4-year-old-girl	CBS1464	2-146	CBS1464			
abscess on epididymis	CBS1227	2-147	CBS1227			Muller 11

tubercular lung	CBS1489	2-148	CBS1489			
faeces of Man	CBS2909	2-150	CBS2909			
faeces of Man	CBS2910	2-151	CBS2910			Muller 11
sputum	CBS2919	2-152	CBS2919			
sputum	CBS4255	2-153	CBS4255			
lung of man with immune deficiency syndrome	CBS7833	**2-154**	CBS7833			Muller 11
substrate of isolation = strain YJM454	CBS7834	**2-155**	CBS7834			
peritoneal fluid, of patient	CBS7835	**2-156**	CBS7835			Muller 11
paracentesis fluid	CBS7836	**2-157**	CBS7836			Muller 11
blood, of patient	CBS7837	**2-158**	CBS7837			Muller 11
patient	CBS7838	**2-159**	CBS7838			Muller 11
bile tube, of patient	CBS7839	**2-160**	CBS7839	Granek 2012 biofilm		Muller 11
ascites fluid	CBS7840	**2-161**	CBS7840			
vaginal fluid	CBS9562	**2-162**	CBS9562			
vaginal fluid	CBS9564	**2-163**	CBS9564			
human	CBS9563	**2-164**	CBS9563			
human	CBS9565	**2-165**	CBS9565			
clinical	GSY723	**2-166**	GSY723		MullerMcC 09	
clinical	GSY725	**2-167**	GSY725	Clemons 94	MullerMcC 09	
single colony isolate from prescription probiotic "Florastor" capsule	GSY1033	**2-168**	GSY1033			
single colony isolate of Saccharomyces from blood of infected 3-year-old who had been taking "Florastor" capsules	GSY1034	**2-169**	GSY1034			
