# Supplementary material for: Whole Genome Analysis of 132 Clinical Saccharomyces cerevisiae Strains Reveals Extensive Ploidy Variation
Source: G3 (Bethesda). 2016 Jun 13;6(8):2421–34. doi: 10.1534/g3.116.029397 (PMC4978896; doi:10.1534/g3.116.029397)
Supplement: Supplemental Material [file supp_g3.116.029397_TableS2.pdf]

Table S2

List of all fragments thought to be introgressed in strains CBS2909 and CBS2910, and genes that fall within these regions.

Chrm	Start	End	Gene	Region	CBS2909	CBS2910
3	86011	86940	YCL009C	85K-115K	Y	Y
3	87096	88253	YCL008C	85K-115K	Y	Y
3	88549	88817	YCL005W-A	85K-115K	Y	Y
3	89419	90189	YCL005W	85K-115K	Y	Y
3	90507	92072	YCL004W	85K-115K	Y	Y
3	92205	93072	YCL002C	85K-115K	Y	Y
3	93315	93881	YCL001W	85K-115K	Y	Y
3	94509	94955	YNL001W	85K-115K	Y	Y
3	95346	95546	YNL001W	85K-115K	Y	Y
3	96097	96210	YLR334C	85K-115K	Y	Y
3	96241	96471	YIL060W	85K-115K	Y	Y
3	99384	100352	YCR002C	85K-115K	Y	Y
3	100636	101187	YCR003W	85K-115K	Y	Y
3	101595	102344	YCR004C	85K-115K	Y	Y
3	102980	104362	YNR001C	85K-115K	Y	Y
3	104665	105054	YCR006C	85K-115K	Y	Y
3	105656	105727	Scer_3.97	85K-115K	Y	Y
3	108158	108301	YOR192C-C	85K-115K	Y	Y
3	108304	109023	YCR007C	85K-115K	Y	Y
3	109728	109838	YLR334C	85K-115K	Y	Y
3	110337	110402	YPR002C-A	85K-115K	Y	Y
3	110390	110590	YDR366C	85K-115K	Y	Y
3	110530	110658	YIL060W	85K-115K	Y	Y
3	110666	110739	Scer_3.102	85K-115K	Y	Y
3	111422	113233	YCR008W	85K-115K	Y	Y
3	113688	114485	YCR009C	85K-115K	Y	Y
3	115238	116089	YCR010C	85K-115K	Y	Y
4	434614	435546	YDL015C	435K-440K	Y	Y
4	435981	436973	YDL014W	435K-440K	Y	Y
4	437704	439551	YDL013W	435K-440K	Y	Y
4	439950	440012	YDL012C	435K-440K	Y	Y
4	440099	440143	YDL012C	435K-440K	Y	Y
4	518452	520788	YDR034C	518K-522K	Y	Y
4	521262	521441	YDR366C	518K-522K	Y	Y
4	521435	521548	YLR334C	518K-522K	Y	Y
4	521759	521842	Scer_4.307	518K-522K	Y	Y
4	555575	556579	YDR051C	555K-570K	Y	Y
4	557035	559137	YDR052C	555K-570K	Y	Y
4	559958	560851	YDR054C	555K-570K	Y	Y
4	562057	563406	YDR055W	555K-570K	Y	Y
4	563566	564183	YDR056C	555K-570K	Y	Y
4	564468	566135	YDR057W	555K-570K	Y	Y
4	566309	567289	YDR058C	555K-570K	Y	Y
4	567491	567562	Scer_4.337	555K-570K	Y	Y

4	567572	567643	Scer_4.338	555K-570K	Y	Y
4	567851	568388	YDR059C	555K-570K	Y	Y
4	569038	569157	YDR366C	555K-570K	Y	Y
4	1009851	1011347	YDR348C	1,010K-1,020K	Y	Y
4	1011595	1012560	YDR347W	1,010K-1,020K	Y	Y
4	1013089	1014540	YDR346C	1,010K-1,020K	Y	Y
4	1017137	1018840	YMR011W	1,010K-1,020K	Y	Y
4	1019403	1019839	YDR344C	1,010K-1,020K	Y	Y
4	1034172	1034276	YNL054W-B	1,032K-1,036K	Y	Y
4	1034277	1035053	YNL054W-B	1,032K-1,036K	Y	Y
4	1035054	1035398	YNL054W-B	1,032K-1,036K	Y	Y
4	1035399	1035944	YNL054W-B	1,032K-1,036K	Y	Y
4	1035991	1036209	YLR334C	1,032K-1,036K	Y	Y
4	1038025	1038189	YOL106W	1,038K-1,042K	Y	Y
4	1038219	1038289	Scer_4.596	1,038K-1,042K	Y	Y
4	1038548	1039363	YDR262W	1,038K-1,042K	Y	Y
4	1039651	1040943	YDR263C	1,038K-1,042K	Y	Y
4	1041410	1043713	YDR264C	1,038K-1,042K	Y	Y
4	1174041	1178582	YDR334W	1,176K-1,189K	Y	Y
4	1179278	1182952	YDR335W	1,176K-1,189K	Y	Y
4	1183219	1184172	YDR336W	1,176K-1,189K	Y	Y
4	1184449	1185309	YDR337W	1,176K-1,189K	Y	Y
4	1185523	1187610	YDR338C	1,176K-1,189K	Y	Y
4	1188113	1188682	YDR339C	1,176K-1,189K	Y	Y
4	1233117	1235048	YDR398W	1,234K-1,239K	Y	Y
4	1235699	1236364	YDR399W	1,234K-1,239K	Y	Y
4	1236690	1237712	YDR400W	1,234K-1,239K	Y	Y
4	1237860	1239329	YDR402C	1,234K-1,239K	Y	Y
4	1455277	1456389	YDR517W	1,456K-1,495K	Y	Y
4	1456660	1458213	YDR518W	1,456K-1,495K	Y	Y
4	1458493	1458900	YDR519W	1,456K-1,495K	Y	Y
4	1459137	1461455	YDR520C	1,456K-1,495K	Y	Y
4	1461836	1463245	YDR522C	1,456K-1,495K	Y	Y
4	1463598	1465070	YDR523C	1,456K-1,495K	Y	Y
4	1465594	1467042	YDR524C	1,456K-1,495K	Y	Y
4	1467964	1468280	YDR525W	1,456K-1,495K	Y	Y
4	1468645	1468881	YDR525W-A	1,456K-1,495K	Y	Y
4	1469134	1470429	YDR527W	1,456K-1,495K	Y	Y
4	1472651	1473916	YDR528W	1,456K-1,495K	Y	Y
4	1474210	1474593	YDR529C	1,456K-1,495K	Y	Y
4	1474837	1475814	YDR530C	1,456K-1,495K	Y	Y
4	1476263	1477342	YDR531W	1,456K-1,495K	Y	Y
4	1477429	1478586	YDR532C	1,456K-1,495K	Y	Y
4	1479356	1480069	YDR533C	1,456K-1,495K	Y	Y
4	1481795	1482429	YDR534C	1,456K-1,495K	Y	Y
4	1482441	1482671	YOR383C	1,456K-1,495K	Y	Y
4	1484517	1484918	YKL078W	1,456K-1,495K	Y	Y
4	1485448	1487262	YDR536W	1,456K-1,495K	Y	Y
4	1488463	1489191	YDR538W	1,456K-1,495K	Y	Y

4	1489637	1491148	YDR539W	1,456K-1,495K	Y	Y
4	1491785	1491916	YOR192C-C	1,456K-1,495K	Y	Y
4	1492677	1493711	YDR541C	1,456K-1,495K	Y	Y
5	173275	173652	YER009W	173K-180K	Y	Y
5	173786	174490	YER010C	173K-180K	Y	Y
5	176432	176968	YER011W	173K-180K	Y	Y
5	176600	178364	YER011W	173K-180K	Y	Y
5	176719	176970	YJR151C	173K-180K	Y	Y
5	178741	178980	YCR018C-A	173K-180K	Y	Y
5	179139	179210	Scer_5.115	173K-180K	Y	Y
5	179895	180491	YER012W	173K-180K	Y	Y
5	273615	274874	YER059W	274K-293K	Y	Y
5	275575	277143	YER060W-A	274K-293K	Y	Y
5	277308	277559	YLR389C	274K-293K	Y	Y
5	277564	279195	YER060W	274K-293K	Y	Y
5	279348	280676	YER061C	274K-293K	Y	Y
5	280978	281730	YER062C	274K-293K	Y	Y
5	282764	283414	YER063W	274K-293K	Y	Y
5	283757	285274	YER064C	274K-293K	Y	Y
5	286256	287929	YER065C	274K-293K	Y	Y
5	289507	289588	Scer_5.179	274K-293K	Y	Y
5	289734	289955	YER181C	274K-293K	Y	Y
5	290132	291292	YFL009W	274K-293K	Y	Y
5	291092	291292	YER066W	274K-293K	Y	Y
5	292113	292490	YER066C-A	274K-293K	Y	Y
5	292527	293012	YER067W	274K-293K	Y	Y
5	303251	303640	YER072W	303K-306K	Y	Y
5	304464	306026	YOR374W	303K-306K	Y	Y
5	322591	322663	Scer_5.193	322K-324K	Y	Y
5	322836	323018	YCR018C-A	322K-324K	Y	Y
5	323155	324063	YER076C	322K-324K	Y	Y
5	519628	522183	YER167W	521K-524K	Y	Y
5	522443	524083	YER168C	521K-524K	Y	Y
6	149251	149862	YFL010C	149K-155K	Y	Y
6	150261	152594	YFL009W	149K-155K	Y	Y
6	153481	157167	YFL008W	149K-155K	Y	Y
6	190130	190200	Scer_6.113	190K-193K	Y	Y
6	190390	192648	YFR009W	190K-193K	Y	Y
6	275395	275766	YFR049W	275K-301K	Y	Y
6	275936	276736	YFR050C	275K-301K	Y	Y
6	277042	278682	YFR051C	275K-301K	Y	Y
6	279372	280196	YFR052W	275K-301K	Y	Y
6	280460	281917	YGL253W	275K-301K	Y	Y
6	283475	285466	YAL063C	275K-301K	Y	Y
6	287749	290547	YFR054C	275K-301K	Y	Y
6	294855	295088	YFR056C	275K-301K	Y	Y
6	295090	296292	YFR055W	275K-301K	Y	Y
6	298657	298788	YBL108W	275K-301K	Y	Y
6	298793	298885	YHL045W	275K-301K	Y	Y

6	298996	299475	YOR394W	275K-301K	Y	Y
6	300332	301690	YHL048W	275K-301K	Y	Y
7	397031	398563	YGL055W	397K-405K	Y	Y
7	399252	399668	YGL054C	397K-405K	Y	Y
7	399908	399979	Scer_7.228	397K-405K	Y	Y
7	400635	400844	YNL054W-B	397K-405K	Y	Y
7	400929	401216	YER181C	397K-405K	Y	Y
7	401645	401716	Scer_7.237	397K-405K	Y	Y
7	401958	402779	YGL050W	397K-405K	Y	Y
7	403036	405780	YGL049C	397K-405K	Y	Y
7	414190	415119	YGL043W	414K-426K	Y	Y
7	415125	415190	YGL042C	414K-426K	Y	Y
7	415232	415300	YGL042C	414K-426K	Y	Y
7	415533	415997	YGL041W-A	414K-426K	Y	Y
7	416228	417256	YGL040C	414K-426K	Y	Y
7	418403	418513	YLR334C	414K-426K	Y	Y
7	418553	418696	YHR145C	414K-426K	Y	Y
7	420013	421056	YGL039W	414K-426K	Y	Y
7	421358	422800	YGL038C	414K-426K	Y	Y
7	423284	423934	YGL037C	414K-426K	Y	Y
7	424598	427300	YGL036W	414K-426K	Y	Y
8	262423	264354	YDR341C	263K-267K	Y	Y
8	264669	266399	YFL011W	263K-267K	Y	Y
8	274872	276098	YHR097C	274K-279K	Y	Y
8	276622	279417	YHR098C	274K-279K	Y	Y
8	432092	432220	YHR175W-A	432K-435K	Y	Y
8	432805	434103	YHR176W	432K-435K	Y	Y
9	232459	232896	YIL051C	232K-236K	Y	Y
9	233523	234380	YIL050W	232K-236K	Y	Y
9	234771	235532	YIL049W	232K-236K	Y	Y
9	243271	245184	YIL046W	241K-245K	Y	Y
9	299445	299516	Scer_9.191	299K-311K	Y	Y
9	299696	299809	YLR334C	299K-311K	Y	Y
9	299800	299922	YPR002C-A	299K-311K	Y	Y
9	299935	300003	YIL060W	299K-311K	Y	Y
9	300160	300480	YIL014C-A	299K-311K	Y	Y
9	300753	300825	Scer_9.197	299K-311K	Y	Y
9	301105	302997	YIL014W	299K-311K	Y	Y
9	303225	307463	YPL058C	299K-311K	Y	Y
9	308120	308398	YIL012W	299K-311K	Y	Y
9	308720	309520	YIL011W	299K-311K	Y	Y
9	309861	310508	YIL010W	299K-311K	Y	Y
9	310648	311191	YIL009C-A	299K-311K	Y	Y
10	89350	91080	YJL172W	90K-99K	Y	Y
10	91292	92482	YJL171C	90K-99K	Y	Y
10	92735	93493	YJL170C	90K-99K	Y	Y
10	93706	93789	YJL169W	90K-99K	Y	Y
10	93786	93809	YJL169W	90K-99K	Y	Y
10	93812	96013	YJL168C	90K-99K	Y	Y

10	96607	97665	YJL167W	90K-99K	Y	Y
10	98031	98312	YJL166W	90K-99K	Y	Y
10	98490	101054	YJL165C	90K-99K	Y	Y
10	397170	398855	YJL016W	397K-399K	Y	Y
10	398859	399098	YJL015C	397K-399K	Y	Y
11	267238	268311	YKL098W	268K-275K	Y	Y
11	269728	270006	YKL096W-A	268K-275K	Y	Y
11	271634	272326	YKL096W	268K-275K	Y	Y
11	272750	273586	YKL095W	268K-275K	Y	Y
11	273815	274756	YKL094W	268K-275K	Y	Y
11	526785	528011	YKR038C	527K-539K	Y	Y
11	528111	528182	Scer_11.295	527K-539K	Y	Y
11	528393	528503	YLR334C	527K-539K	Y	Y
11	528572	528676	YLR334C	527K-539K	Y	Y
11	528665	528862	YER181C	527K-539K	Y	Y
11	529966	531774	YKR039W	527K-539K	Y	Y
11	532592	532664	Scer_11.303	527K-539K	Y	Y
11	532777	533535	YKR041W	527K-539K	Y	Y
11	534117	535199	YKR042W	527K-539K	Y	Y
11	535489	536304	YKR043C	527K-539K	Y	Y
11	536604	537935	YKR044W	527K-539K	Y	Y
11	538007	538549	YKR045C	527K-539K	Y	Y
11	538782	539633	YKR046C	527K-539K	Y	Y
13	325518	327527	YMR032W	326K-330K	Y	Y
13	327533	327776	YMR030W-A	326K-330K	Y	Y
13	327998	329488	YMR033W	326K-330K	Y	Y
13	329632	330936	YMR034C	326K-330K	Y	Y
13	488704	489318	YLR029C	488K-498K	Y	Y
13	489661	490023	YMR122C	488K-498K	Y	Y
13	490423	490506	YMR122W-A	488K-498K	Y	Y
13	492235	492450	YMR122W-A	488K-498K	Y	Y
13	492780	493148	YMR273C	488K-498K	Y	Y
13	493644	496460	YMR124W	488K-498K	Y	Y
13	496711	499618	YMR125W	488K-498K	Y	Y
13	595251	599507	YMR176W	597K-609K	Y	Y
13	600111	601640	YMR177W	597K-609K	Y	Y
13	602008	602832	YMR178W	597K-609K	Y	Y
13	603389	605668	YMR179W	597K-609K	Y	Y
13	605766	606728	YMR180C	597K-609K	Y	Y
13	607133	607594	YMR181C	597K-609K	Y	Y
13	608047	608685	YMR182C	597K-609K	Y	Y
14	218097	219398	YNL220W	218K-220K	Y	Y
15	273097	273222	YOL019W-A	273K-275K	Y	Y
15	273546	275207	YOL019W	273K-275K	Y	Y
15	902768	907765	YOR341W	902K-917K	Y	Y
15	908436	909395	YOR342C	902K-917K	Y	Y
15	909910	910207	YOR343C	902K-917K	Y	Y
15	910636	910728	YDR366C	902K-917K	Y	Y
15	911367	911498	YNL054W-B	902K-917K	Y	Y

15	911521	911577	YDR366C	902K-917K	Y	Y
15	911560	911655	YHR145C	902K-917K	Y	Y
15	912208	912280	Scer_15.585	902K-917K	Y	Y
15	912984	913859	YOR344C	902K-917K	Y	Y
15	916157	916228	YOR192C-C	902K-917K	Y	Y
15	916607	916711	Scer_15.591	902K-917K	Y	Y
16	830545	831288	YPR143W	830K-853K	Y	Y
16	831751	831824	Scer_16.481	830K-853K	Y	Y
16	831972	833630	YPR144C	830K-853K	Y	Y
16	834827	836545	YGR124W	830K-853K	Y	Y
16	836892	837125	YPR145C-A	830K-853K	Y	Y
16	837576	837779	YPR146C	830K-853K	Y	Y
16	837855	838769	YPR147C	830K-853K	Y	Y
16	839060	840346	YPR148C	830K-853K	Y	Y
16	842119	842640	YPR149W	830K-853K	Y	Y
16	843233	843847	YPR151C	830K-853K	Y	Y
16	844226	845590	YPR152C	830K-853K	Y	Y
16	845967	846371	YPR153W	830K-853K	Y	Y
16	846692	847324	YPR154W	830K-853K	Y	Y
16	847635	849485	YPR155C	830K-853K	Y	Y
16	849958	851826	YGR138C	830K-853K	Y	Y
5	524791	527181	YER169W	524K-534K		Y
5	527391	528098	YER170W	524K-534K		Y
5	528507	530837	YER171W	524K-534K		Y
5	563715	564632	YER186C	563K-574K		Y
5	567443	568666	YER187W	563K-574K		Y
5	569795	570265	YER188W	563K-574K		Y
5	570289	570330	YPR202W	563K-574K		Y
5	570339	570467	YNL339C	563K-574K		Y
5	570468	570857	YLR467W	563K-574K		Y
5	570858	571091	YBL111C	563K-574K		Y
5	571093	571947	YER190W	563K-574K		Y
5	571963	572724	YPL283C	563K-574K		Y
5	572872	574002	YPL283C	563K-574K		Y
4	328918	331020	YDL074C	330K-335K	Y	
4	331487	334447	YDL073W	330K-335K	Y	
4	334698	335309	YDL072C	330K-335K	Y	
4	466527	469022	YDR006C	466K-479K	Y	
4	469826	470500	YDR007W	466K-479K	Y	
4	471261	472823	YBR020W	466K-479K	Y	
4	473778	478283	YOR011W	466K-479K	Y	
6	202184	204310	YLR258W	202K-204K	Y	
7	448955	451219	YGL022W	450K-455K	Y	
7	451523	453730	YGL021W	450K-455K	Y	
7	453826	454533	YGL020C	450K-455K	Y	
7	454818	455654	YGL019W	450K-455K	Y	
15	111272	112210	YOL101C	111K-123K	Y	
15	113542	116790	YOL100W	111K-123K	Y	
15	117028	120141	YOL098C	111K-123K	Y	

15	120542	120718	YOL097W-A	111K-123K	Y
15	120841	122139	YOL097C	111K-123K	Y
15	122410	123348	YOL096C	111K-123K	Y
