# Supplementary material for: Whole Genome Analysis of 132 Clinical Saccharomyces cerevisiae Strains Reveals Extensive Ploidy Variation
Source: G3 (Bethesda). 2016 Jun 13;6(8):2421–34. doi: 10.1534/g3.116.029397 (PMC4978896; doi:10.1534/g3.116.029397)
Supplement: Supplemental Material [file supp_g3.116.029397_TableS4.pdf]

Table S4: Lists of all genes that showed complete deletion

Frequency	Gene
1	*SEO1*
1	*YAT1*
1	*COS2*
1	*RDS1*
1	*YER134C*
1	*SNZ3*
1	*SNO3*
1	*PAU13*
1	*COS8*
1	*PHO12*
1	*IMA5*
1	*DAL5*
1	*PGU1*
1	*FLO10*
1	*SNZ2*
1	*SNO2*
1	*PAU6*
1	*IMA2*
1	*PAU20*
1	*HSP33*
1	*ARR3*
2	*PHO11*
2	*PAU3*
2	*HXT9*
2	*YLR162W*
2	*PAU19*
2	*YOL159C*
2	*FIT2*
2	*FIT3*
2	*ERR2*
3	*MST28*
3	*DAK2*
3	*NFT1*
3	*AYT1*
3	*COS10*
3	*HXT11*
3	*YOL159C-A*
4	*ADH7*
4	*MAL13*
4	*ARN2*
4	*YIL060W*
5	*VBA3*
5	*COS1*
6	*UIP3*

6	*MATALPHA1*
6	*THI5*
6	*VBA5*
6	*ERR1*
7	*GEX1*
7	*HXT15*
7	*SNO4*
7	*YPR204W*
8	**PRM9**
8	**AGP3**
8	**MAL11**
8	**YKR104W**
10	**MST27**
10	**GEX2**
10	**YRF1-4**
11	**CUP1-1**
11	**AIF1**
14	**PRM8**
15	**AAD16**
17	**PAU16**
18	**MPH3**
23	**VTH2**
24	**SOR1**
29	**REE1**
31	**YRF1-2**
32	**PAU15**
44	**BDS1**
47	**AAD15**
50	**YAL064W**
53	**MPH2**
59	**CUP1-2**
69	**PAU21**
70	**PAU18**
86	**YBL111C**
86	**ENA2**
86	**HXT16**
90	VTH1
92	IMA4
92	PAU22
93	YRF1-1
93	YRF1-3
93	IMA3
93	PAU1
93	TAR1
93	ASP3-1
93	ASP3-2

93	ASP3-3
93	ASP3-4
93	YRF1-5
93	YRF1-6
93	YRF1-7

Genes names in *underlined italics* or **bold** represent those that were classified as *rare* or **common** respectively.
