# Supplementary material for: Whole Genome Analysis of 132 Clinical Saccharomyces cerevisiae Strains Reveals Extensive Ploidy Variation
Source: G3 (Bethesda). 2016 Jun 13;6(8):2421–34. doi: 10.1534/g3.116.029397 (PMC4978896; doi:10.1534/g3.116.029397)
Supplement: Supplemental Material [file supp_g3.116.029397_TableS5.pdf]

Table S5: List of all genes that showed copy number gain

Count	Gene	Symbol	Name
1	YAR071W	*PHO11*	PHOsphate metabolism
1	YCL073C	*GEX1*	Glutathione EXchanger
1	YDL244W	*THI13*	THIamine metabolism
1	YEL069C	*HXT13*	HeXose Transporter
1	YGL201C	*MCM6*	MiniChromosome Maintenance
1	YGL202W	*ARO8*	AROmatic amino acid requiring
1	YGR088W	*CTT1*	CaTalase T
1	YGR203W	*YCH1*	Yeast Cdc25 Homologue
1	YHL028W	*WSC4*	cell Wall integrity and Stress response Component
1	YHL043W	*ECM34*	ExtraCellular Mutant
1	YHR044C	*DOG1*	DeOxyGlucose
1	YHR055C	*CUP1-2*	
1	YJL011C	*RPC17*	RNA Polymerase C
1	YJL012C	*VTC4*	Vacuolar Transporter Chaperone
1	YJL013C	*MAD3*	Mitotic Arrest-Deficient
1	YJL014W	*CCT3*	Chaperonin Containing TCP-1
1	YJL019W	*MPS3*	MonoPolar Spindle
1	YJL020C	*BBC1*	Bni1 synthetic lethal and Bee1 (las17) Complex member
1	YJL024C	*APS3*	clathrin Associated Protein complex Small subunit
1	YJL025W	*RRN7*	Regulation of RNA polymerase I
1	YJL026W	*RNR2*	RiboNucleotide Reductase
1	YJL028W	*YJL028W*	
1	YJL029C	*VPS53*	Vacuolar Protein Sorting
1	YJL030W	*MAD2*	Mitotic Arrest-Deficient
1	YJL031C	*BET4*	Blocked Early in Transport
1	YJL033W	*HCA4*	Helicase CA
1	YJL034W	*KAR2*	KARyogamy
1	YJL035C	*TAD2*	tRNA-specific Adenosine Deaminase
1	YJL036W	*SNX4*	Sorting NeXin
1	YJL037W	*IRC18*	Increased Recombination Centers
1	YJL038C	*LOH1*	Loss Of Heterozygosity
1	YJL039C	*NUP192*	NUclear Pore
1	YJL041W	*NSP1*	NucleoSkeletal-like Protein
1	YJL042W	*MHP1*	MAP-Homologous Protein
1	YJL044C	*GYP6*	Gtpase-activating protein of Ypt6 Protein
1	YJL045W	*YJL045W*	
1	YKL029C	*MAE1*	MAlic Enzyme
1	YKL198C	*PTK1*	Putative serine/Threonine protein Kinase
1	YKL219W	*COS9*	COnserved Sequence
1	YLR303W	*MET17*	METhionine requiring
1	YLR385C	*SWC7*	SWr Complex
1	YMR042W	*ARG80*	ARGinine requiring
1	YMR323W	*ERR3*	Enolase-Related Repeat
1	YNL334C	*SNO2*	SNZ proximal Open reading frame
1	YNR074C	*AIF1*	Apoptosis-Inducing Factor
1	YNR076W	*PAU6*	seriPAUperin family
1	YOL155C	*HPF1*	Haze Protective Factor

1	YOL164W	*BDS1*	Bacterially Derived Sulfatase
1	YOR209C	*NPT1*	Nicotinate PhosphoribosylTransferase
1	YOR285W	*RDL1*	RhoDanese-Like protein
1	YOR286W	*RDL2*	RhoDanese-Like protein
1	YOR391C	*HSP33*	Heat-Shock Protein
1	YPL113C	*YPL113C*	
1	YPL279C	*FEX2*	
1	YPR121W	*THI22*	THIamine metabolism
1	YPR193C	*HPA2*	Histone and other Protein Acetyltransferase
2	YBR301W	*PAU24*	seriPAUperin family
2	YFR034C	*PHO4*	PHOsphate metabolism
2	YKL218C	*SRY1*	Serine Racemase of Yeast
2	YLR141W	*RRN5*	Regulation of RNA polymerase I
2	YMR325W	*PAU19*	seriPAUperin
2	YOL110W	*SHR5*	Suppressor of Hyperactive Ras
2	YOR390W	*FEX1*	
3	YAL068C	**PAU8**	seriPAUperin
3	YEL071W	**DLD3**	D-Lactate Dehydrogenase
3	YEL072W	**RMD6**	Required for Meiotic nuclear Division
3	YFL059W	**SNZ3**	SNooZe
3	YKL216W	**URA1**	URAcil requiring
3	YKL217W	**JEN1**	
3	YKL220C	**FRE2**	Ferric REductase
3	YNR075W	**COS10**	COnserved Sequence
3	YOR348C	**PUT4**	Proline UTilization
3	YPR194C	**OPT2**	OligoPeptide Transporter
4	YAL039C	**CYC3**	CYtochrome C
4	YKL215C	**OXP1**	OXoProlinase
4	YLR461W	**PAU4**	seriPAUperin family
4	YNR069C	**BSC5**	Bypass of Stop Codon
4	YNR070W	**PDR18**	Pleiotropic Drug Resistance
4	YOR388C	**FDH1**	
5	YJL214W	**HXT8**	HeXose Transporter
5	YKL222C	**YKL222C**	
5	YPR200C	**ARR2**	ARsenicals Resistance
6	YNR067C	**DSE4**	Daughter Specific Expression
8	YBR299W	**MAL32**	MALtose fermentation
8	YCR104W	**PAU3**	seriPAUperin family
8	YHR215W	**PHO12**	PHOsphate metabolism
8	YKL221W	**MCH2**	MonoCarboxylate transporter Homologue
8	YPR199C	**ARR1**	ARsenicals Resistance
9	YPR198W	**SGE1**	Suppression of Gal11 Expression
10	YPR201W	**ARR3**	ARsenicals Resistance
11	YFL053W	**DAK2**	DihydroxyAcetone Kinase
11	YHR053C	**CUP1-1**	
12	YPL273W	**SAM4**	S-AdenosylMethionine metabolism
13	YFL055W	**AGP3**	high-Affinity Glutamine Permease
14	YCR106W	**RDS1**	Regulator of Drug Sensitivity
15	YGR287C	**IMA1**	IsoMAltase

18	YCR105W	**ADH7**	Alcohol DeHydrogenase
18	YCR107W	**AAD3**	Aryl-Alcohol Dehydrogenase
20	YOL159C	**YOL159C**	
21	YOL158C	**ENB1**	ENteroBactin
21	YOL159C-A	**YOL159C-A**	
24	YAL067C	**SEO1**	Suppressor of sulfoxyde EthiOnine resistance
30	YJR156C	**THI11**	THIamine metabolism
31	YIL162W	**SUC2**	SUCrose fermentation
34	YGL263W	**COS12**	COnserved Sequence
61	YBR298C	**MAL31**	MALtose fermentation
64	YPL274W	**SAM3**	S-AdenosylMethionine metabolism
67	YHR216W	**IMD2**	IMP Dehydrogenase
83	YBL108C-A	PAU9	seriPAUperin

Genes names in *underlined italics* or **bold** represent those that were classified as *rare* or **common** respectively.
