# Supplementary material for: Whole Genome Analysis of 132 Clinical Saccharomyces cerevisiae Strains Reveals Extensive Ploidy Variation
Source: G3 (Bethesda). 2016 Jun 13;6(8):2421–34. doi: 10.1534/g3.116.029397 (PMC4978896; doi:10.1534/g3.116.029397)
Supplement: Supplemental Material [file supp_g3.116.029397_TableS6.pdf]

Table S6: List of all genes that showed copy number loss

Count	Gene	Symbol	Name
1	YGL263W	*COS12*	COnserved Sequence
1	YHR053C	*CUP1-1*	
1	YJR152W	*DAL5*	Degradation of Allantoin
1	YMR323W	*ERR3*	Enolase-Related Repeat
1	YOR381W	*FRE3*	Ferric REductase
1	YBR187W	*GDT1*	Gcr1 Dependent Translation factor
1	YOL156W	*HXT11*	HeXose Transporter
1	YJL221C	*IMA4*	IsoMAltase
1	YJL216C	*IMA5*	IsoMAltase
1	YHR216W	*IMD2*	IMP Dehydrogenase
1	YFR055W	*IRC7*	Increased Recombination Centers
1	YBR188C	*NTC20*	Prp19p (NineTeen)-associated Complex
1	YOL161C	*PAU20*	seriPAUperin
1	YPL282C	*PAU22*	seriPAUperin
1	YLR461W	*PAU4*	seriPAUperin family
1	YNR076W	*PAU6*	seriPAUperin family
1	YJR153W	*PGU1*	PolyGalactUronase
1	YBR092C	*PHO3*	PHOsphate metabolism
1	YOR386W	*PHR1*	PHotoreactivation Repair deficient
1	YMR266W	*RSN1*	Rescue of Sro7 at high Nacl
1	YNL333W	*SNZ2*	SNooZe
2	YJR155W	*AAD10*	Aryl-Alcohol Dehydrogenase
2	YMR170C	*ALD2*	ALdehyde Dehydrogenase
2	YOR383C	*FIT3*	Facilitator of Iron Transport
2	YAR050W	*FLO1*	FLOcculation
2	YJL214W	*HXT8*	HeXose Transporter
2	YJL219W	*HXT9*	HeXose Transporter
2	YOL157C	*IMA2*	IsoMAltase
2	YMR325W	*PAU19*	seriPAUperin
2	YCR104W	*PAU3*	seriPAUperin family
2	YAR071W	*PHO11*	PHOsphate metabolism
2	YHR215W	*PHO12*	PHOsphate metabolism
2	YBR294W	*SUL1*	SULfate metabolism
2	YFL058W	*THI5*	THIamine metabolism
2	YOL159C	*YOL159C*	
3	YLL063C	**AYT1**	AcetYlTransferase
3	YOR382W	**FIT2**	Facilitator of Iron Transport
3	YCR098C	**GIT1**	GlycerophosphoInosiTol
3	YBR295W	**PCA1**	P-type Cation-transporting ATPase
3	YDL244W	**THI13**	THIamine metabolism
3	YIL173W	**VTH1**	
4	YNR074C	**AIF1**	Apoptosis-Inducing Factor
4	YDL248W	**COS7**	COnserved Sequence
4	YHL048W	**COS8**	COnserved Sequence

4	YGR292W	**MAL12**	MALtose fermentation
4	YPR121W	**THI22**	THIamine metabolism
4	YBL111C	**YBL111C**	
4	YOL159C-A	**YOL159C-A**	
5	YCR105W	**ADH7**	Alcohol DeHydrogenase
5	YHL047C	**ARN2**	AFT1 ReguloN
5	YMR244C-A	**COA6**	Cytochrome Oxidase Assembly
5	YOL158C	**ENB1**	ENteroBactin
5	YOR384W	**FRE5**	Ferric REductase
5	YAL067C	**SEO1**	Suppressor of sulfoxyde EthiOnine resistance
6	YHR055C	**CUP1-2**	
6	YJR158W	**HXT16**	HeXose Transporter
6	YFL060C	**SNO3**	SNZ proximal Open reading frame
6	YJR159W	**SOR1**	
7	YGR295C	**COS6**	COnserved Sequence
7	YDR036C	**EHD3**	
7	YDR039C	**ENA2**	Exitus NAtru (Latin
7	YCL073C	**GEX1**	Glutathione EXchanger
8	YCR107W	**AAD3**	Aryl-Alcohol Dehydrogenase
8	YDR038C	**ENA5**	Exitus NAtru (Latin
8	YGL051W	**MST27**	Multicopy suppressor of Sec Twenty one
8	YAR031W	**PRM9**	Pheromone-Regulated Membrane protein
8	YCR106W	**RDS1**	Regulator of Drug Sensitivity
8	YCL069W	**VBA3**	Vacuolar Basic Amino acid transporter
9	YNR075W	**COS10**	COnserved Sequence
9	YAR033W	**MST28**	Multicopy suppressor of Sec Twenty one
9	YLL064C	**PAU18**	seriPAUperin
9	YKR105C	**VBA5**	Vacuolar Basic Amino acid transporter
11	YNL336W	**COS1**	COnserved Sequence
13	YOR394W	**PAU21**	seriPAUperin
13	YAL064W	**YAL064W**	
14	YKL170W	**MRPL38**	Mitochondrial Ribosomal Protein
14	YNL332W	**THI12**	THIamine metabolism
16	YFL055W	**AGP3**	high-Affinity Glutamine Permease
16	YDL245C	**HXT15**	HeXose Transporter
16	YHL046C	**PAU13**	seriPAUperin
18	YOL165C	**AAD15**	Aryl-Alcohol Dehydrogenase
18	YML132W	**COS3**	COnserved Sequence
18	YKR106W	**GEX2**	Glutathione EXchanger
18	YJL217W	**REE1**	REgulation of Enolase
19	YDR040C	**ENA1**	Exitus NAtru (Latin
19	YIR041W	**PAU15**	seriPAUperin
19	YMR322C	**SNO4**	SNZ proximal Open reading frame

20	YPL143W	**RPL33A**	Ribosomal Protein of the Large subunit
21	YFL053W	**DAK2**	DihydroxyAcetone Kinase
21	YJR160C	**MPH3**	Maltose Permease Homolog
21	YAR027W	**UIP3**	Ulp1 Interacting Protein
21	YKR104W	**YKR104W**	
23	YGR289C	**MAL11**	MALtose fermentation
24	YBR302C	**COS2**	COnserved Sequence
25	YKR102W	**FLO10**	FLOcculation
25	YGR288W	**MAL13**	MALtose fermentation
25	YGL103W	**RPL28**	Ribosomal Protein of the Large subunit
26	YKR103W	**NFT1**	New Full-length MRP-type Transporter
27	YOR393W	**ERR1**	Enolase-Related Repeat
29	YFL057C	**AAD16**	Aryl Alcohol Dehydrogenase
30	YPR204W	**YPR204W**	
31	YDL247W	**MPH2**	Maltose Permease Homolog
33	YFL056C	**AAD6**	Aryl-Alcohol Dehydrogenase
33	YOL164W	**BDS1**	Bacterially Derived Sulfatase
33	YPL281C	**ERR2**	Enolase-Related Repeat
35	YKL224C	**PAU16**	seriPAUperin
35	YLR466W	**YRF1-4**	
36	YGL053W	**PRM8**	Pheromone-Regulated Membrane protein
37	YJL222W	**VTH2**	
38	YIL060W	**YIL060W**	
41	YCR040W	**MATALPHA1**	MATing type protein ALPHA
43	YER190W	**YRF1-2**	
44	YPL249C-A	**RPL36B**	Ribosomal Protein of the Large subunit
53	YJR022W	**LSM8**	Like SM
56	YLR162W	**YLR162W**	
66	YJL189W	**RPL39**	Ribosomal Protein of the Large subunit
68	YLR061W	**RPL22A**	Ribosomal Protein of the Large subunit
72	YIR017C	MET28	METhionine requiring
76	YPR043W	RPL43A	Ribosomal Protein of the Large subunit

Genes names in <u>*underlined italics*</u> or **bold** represent those that were classified as <u>*rare*</u> or **common** respectively.
